# *Orientia tsutsugamushi* in chiggers and small mammals in Laos

**DOI:** 10.1089/vbz.2022.0029

**Published:** 2022-10-06

**Authors:** Ivo Elliott, Rawadee Kumlert, Neeranuch Thangnimitchok, Stuart D. Blacksell, Ampai Tanganuchitcharnchai, Daniel H. Paris, Paul N. Newton, Serge Morand

**Affiliations:** 1 Lao-Oxford-Mahosot Hospital-Wellcome Trust Research Unit, Microbiology Laboratory, Mahosot Hospital, Vientiane, Lao PDR; 2 Centre for Tropical Medicine and Global Health, Nuffield Department of Medicine, University of Oxford, Oxford, United Kingdom; 3 Division of Vector-Borne Diseases, Department of Disease Control, Ministry of Public Health, Nonthaburi, Thailand; 4 Mahidol-Oxford Tropical Medicine Research Unit, Faculty of Tropical Medicine, Mahidol University, Bangkok, Thailand; 5 Department of Medicine, Swiss Tropical and Public Health Institute, Basel, Switzerland; 6 Department of Clinical Research, University of Basel, Basel, Switzerland; 7 MIVEGEC, CNRS, IRD, Montpellier University, Montpellier, France; 8 Faculty of Veterinary Technology, Kasetsart University, Bangkok, Thailand

**Keywords:** scrub typhus, *Orientia tsutsugamushi*, chiggers, Laos

## Abstract

Scrub typhus is a leading cause of febrile illness in Laos and accounts for a high burden of disease. There have been no previous studies on the causative agent, *Orientia tsutsugamushi*, in vector mites (“chiggers”) or their small mammal hosts in Laos. Small mammals and free-living chiggers were trapped in districts of Vientiane Province and Capital. Tissues were tested for *O*. *tsutsugamushi* by PCR and serum for IgG to *O*. *tsutsugamushi* by immunofluorescence assays (IFA). Chiggers removed from small mammals and collected in their free-living stage using black plates were identified and tested for *O*. *tsutsugamushi* by PCR. Over an 18-month period 131 small mammals of 14 species were collected in 5 districts. Seventy-eight of 131 small mammals were infested with chiggers, but all tissues were *O*. *tsutsugamushi* PCR negative. Eighteen species of chigger were identified and 1,609 were tested by PCR. A single pool of chiggers tested *O*. *tsutsugamushi* positive. Serum from 52 small mammals were tested by IFA, with 16 testing positive. These are the first molecular and serological data on *O*. *tsutsugamushi* in chiggers and small mammals in Laos. Further studies are needed to better understand the key vector species and ecology of scrub typhus in areas with high disease incidence in Laos.

## Introduction

Scrub typhus is a potentially fatal neglected tropical disease caused by the obligate intracellular bacterium *Orientia tsutsugamushi*. The pathogen is transmitted by the bite of larval stage trombiculid mites (chiggers) that are predominantly hosted by small mammals[1]. The disease is endemic to the “tsutsugamushi triangle” region of the Asia-Pacific, and threatens over a billion people, leading to severe febrile illness, with complications including pneumonitis, meningoencephalitis, myocarditis and renal failure[2]. The median untreated mortality is ˜6%[3]. Rural workers and military personnel are typically at highest risk, but cases are increasing among city dwellers visiting the countryside for recreation[4].

The Lao PDR (Laos) is a lower-middle income, land-linked country in Southeast Asia with a population of close to 7 million people. Two thirds of the population live in rural areas and half of these are subsistence farmers[5]. Scrub typhus was first reported in Laos in 1939[6], but not subsequently reported until 2006[7]. The disease is a leading cause of febrile illness in Laos, accounting for 14.8% of admissions with community-acquired septicaemia to hospital in the capital, Vientiane[7]. Seroprevalence in the capital was 20% and is likely to be higher in more rural populations[8]. Spatiotemporal analysis of scrub typhus showed a negative association with the more urbanized parts of the capital city and a strong seasonal pattern, with cases peaking in the wettest months of July-September[9].

For the first time in Laos, we investigate for the presence of *O*. *tsutsugamushi* in chiggers and small mammal hosts at sites of high-disease transmission across Vientiane Province. We describe the species composition, dynamics and rates of *O*. *tsutsugamushi* infection in chiggers and small mammals and relate these data to studies performed in the region.

## Materials and methods

### Study sites

Four districts of Vientiane Province and one district of Vientiane Capital, Lao PDR were selected for investigation of small mammals and chiggers. These were selected due to a high incidence of scrub typhus[9] and recently identified scrub typhus cases with evidence of exposure in peri-village habitats. Small mammal trapping took place at several locations in each district, selected and performed by local hunters experienced in trapping small mammals.

### Small mammal and chigger collection

Small mammals were trapped alive using locally made wire-mesh traps baited with a range of food items by local hunters. Small mammal trapping took place in the hot, dry season of May 2015, cool dry season of February 2016 and wet season of October 2016.

Free-living chiggers were collected using the black plate method[10, 11]. Black acrylic plates (10 x 30 cm) were placed on the ground for 10 minutes and examined using a 30-40x hand lens for chiggers every 2 minutes. Chiggers were collected using a pipette and transferred into a sterile tube containing 70% ethanol. The location of all collected field samples were recorded using a Garmin Oregon (Garmin International, Olathe, Kansas) GPS device (accurate to ~3 metres).

### Small mammal and chigger identification and processing

Traps containing small mammals were transferred to a mobile laboratory and placed in an airtight box with the addition of cotton wool soaked with 10 ml of the inhalational anaesthetic isoflurane. Animals were only removed from the trap once dead. All animal handling and euthanasia procedures followed international guidelines[12, 13]. All field and mobile laboratory protocols and procedures followed the “Protocols for field and laboratory rodent studies” 2011 guide[14]. This study was approved by the Oxford Tropical Research Ethics Committee (OxTREC 48-15 & 52-14) and by the National Health Ethics Committee, Laos (039/2016/NEHCR).

The methods used follow those described in Elliott et al[15]. In brief, small mammals were weighed in grams and the head and body, tail, hind foot, ear and skull measured in millimetres and identified to species level wherever possible following the keys in Chaval, 2011[16]. Prior to harvesting tissues, scissors and forceps were thoroughly washed consecutively in Dettol (chloroxylenol), sterile water and 70% ethanol to prevent cross-contamination. Small pieces (<50 mg) of lung, liver and spleen were collected into pre-labelled cryotubes and stored on dry ice in the field and subsequently at -80°C in the laboratory.

Both ears were detached as close to the skull as possible and stored in 70% ethanol at +4°C. The rest of the animal was examined for chiggers, notably the posterior legs, ventral midline and anogenital region. In the laboratory the ears or skin were examined under a Brunel IMXZ stereo microscope (Brunel Microscopes Ltd., Chippenham, U.K.) at 15x to 100x magnification. Approximately 15% of chiggers on each animal were identified to species. These were selected based on different appearances under lower-power microscopy in an attempt to capture the diversity of species present on the host. Chiggers were placed between two cover slips on a glass slide and morphological identification to species was performed using a Nikon Eclipse 80i compound microscope at 400x or 600x. Images were viewed and saved using the Nikon NIS Elements D 4.13.05 software package. Both autofluorescence and bright-field microscopy techniques were used[17]. A scale bar was applied to each image. All morphometric measurements and image manipulation was performed using ImageJ (https://imagej.net/ImageJ). The genus was identified with reference to Nadchatram & Dohany 1974[18] and Vercammen-Grandjean 1968 keys to Southeast Asian chigger genera[19]. A wide range of taxonomic identification keys were employed to identify to species level[20–23].

### Small mammal and chigger DNA extraction and PCR

DNA was extracted from individual chiggers, pools of chiggers (~20-50 unidentified individuals of possible multispecies from the same host animal) and rodent tissues using the Qiagen Blood and Tissue Kit (Qiagen, USA). Chiggers were rinsed with distilled water and individuals cut through the mid-gut using a sterile 30G needle under a dissecting microscope. Pools were crushed using a sterile polypropylene motorized pestle (motorized pellet pestle Z35991, Sigma Aldrich, St Louis, MO). Rodent tissues were cut into a small piece (≤10mg of spleen or ≤25mg of liver or lung). Samples were incubated with proteinase K at 56°C for 3 hours. The rest of the steps followed the manufacturer’s protocol. Chigger samples were eluted in 45μl and rodent samples in 100μl of buffer AE (Qiagen, Hilden, Germany). Samples were stored at -20°C before PCR. Real-time PCR targeting the 47kDa *O*. *tsutsugamushi* outer-membrane protein was performed on all samples as previously described[24], except for chigger samples where 5 μl of DNA template was added. PCR was run on a Bio Rad CFX96 (Bio Rad, USA).

### Small mammal serology

The Scrub typhus “gold standard” immunofluorescent assay (IFA) was used to determine the endpoint IgG antibody titer in the rodent samples with the full methodology described elsewhere[25]. Briefly, 40-well slides, produced in-house at the Mahidol-Oxford Tropical Medicine Research Unit (MORU), Bangkok, Thailand, were coated and fixed with type-specific *O*. *tsutsugamushi* antigen strains (Karp, Kato, Gilliam, and TA716). Two-fold serial dilutions of rodent serum from 1:16 to 1:4096 were prepared in 2% skim milk in phosphate buffered saline (PBS) diluent. 2 μl of diluted serum was dropped onto each well and incubated at 37°C for 30 minutes. The slide was then washed four times with PBS, dried and 2 μl of diluted FITC anti-mouse conjugate added to each well. The incubation and washing steps were then repeated and fluorescence mounting medium added to the slide. Two experienced technicians examined the slides to determine the end-point titre as the highest titer displaying specific fluorescence. Samples with a titer ≥1:16 were considered positive for the presence of IgG antibodies[26, 27].

## Results

**Figure 1:**
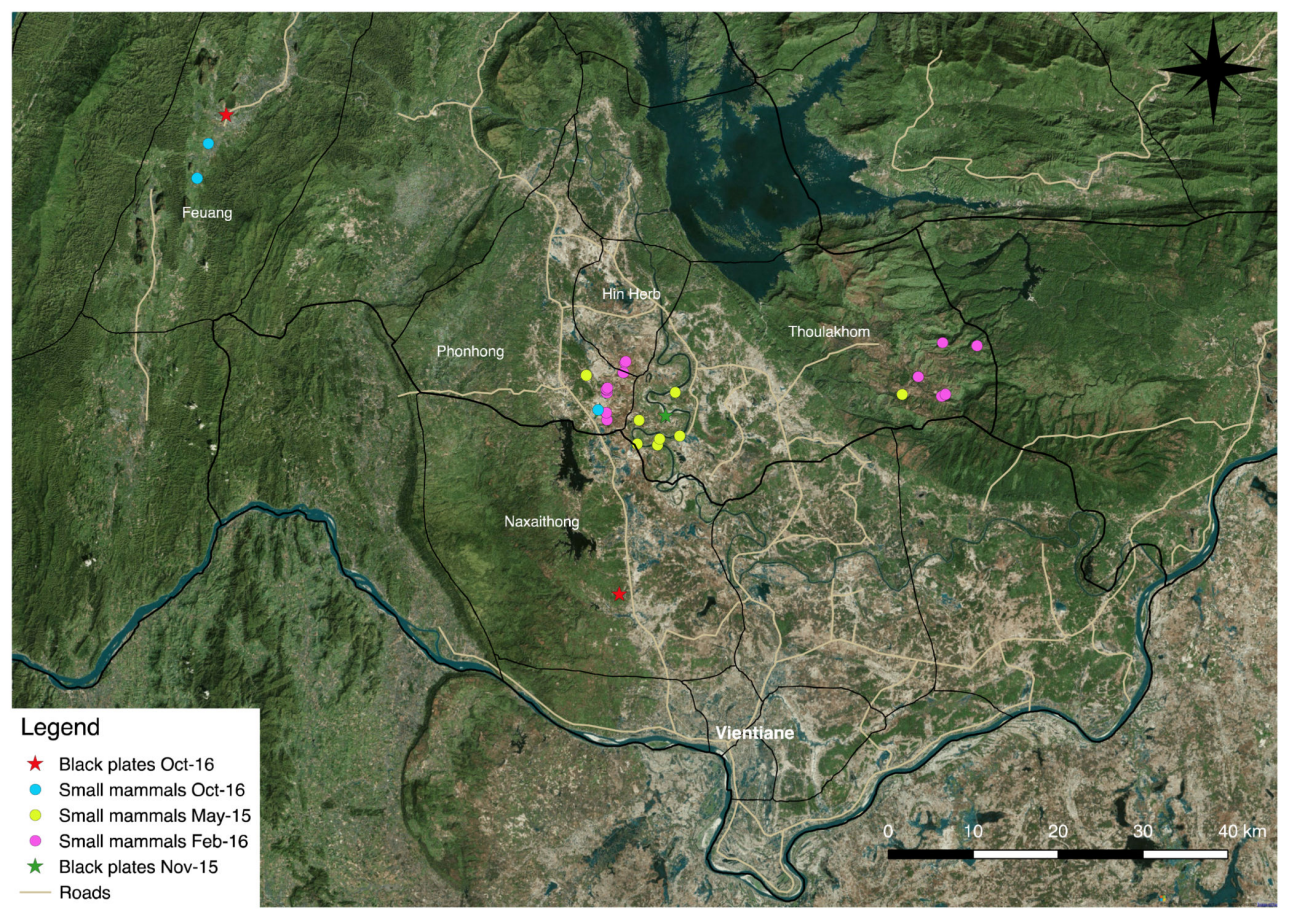
Map showing study sites across Vientiane Province and Vientiane Capital, Lao PDR.

Four districts of Vientiane Province were visited. Small mammals were trapped at 10 sites in Phonehong (18.50° N, 102.42° E), 12 sites across Thoulakhom (18.37° N, 102.74° E), 2 sites in Hin Herb (18.63° N, 102.33° E) and 2 sites in Feuang (18.65° N, 102.09° E) (Figure 1). Sites in eastern Thoulakhom were in upland terrain between 600 and 850 metres above mean sea level (msl). The habitat consists of upland rice fields, fallow areas, bamboo and degraded secondary forest. All other sites were in lowland areas between 170 and 250m above msl, formed of lowland rice paddies, fallow areas and fruit plantations.

A total of 131 small mammals were collected during 3 visits over 18 months. These comprised 14 species of Rodentia and 1 species of Scandentia (*Tupaia glis*) (Table 1). The rice-field specialist *Bandicota indica* was most frequently collected, 33/131 (25%), followed by *Rattus exulans*, 24/131 (18%) and *Niviventer* sp. 15/131 (11%). Due to taxonomic uncertainty associated with juvenile individuals, 14 *Niviventer* and 3 *Maxomys* could only be identified to genus.

Seventy-eight of 131 (60%) individual small mammals were infested with chiggers. *B*. *indica* were frequently infested with chiggers at 28/33 (85%). *R*. *exulans* and *M*. *caroli* were both less frequently infested at 3/24 (13%) and 5/11 (46%), respectively (Table 1). Infestation rates were high for many other species, although numbers of individuals were low.

Small mammals trapped in May 2015 were tested for *O*. *tsutsugamushi* by PCR only from spleen tissue. None tested positive from 71 individuals. The remaining 60 individuals had spleen, liver and lung tested and all tested negative (Table 1).

Of 1,446 individual chiggers removed from rodents, 243 (17%) were identified, composed of 16 species in 8 genera (Table 2). *Walchia* spp. were most frequent with 158/243 (65%) in 8 species; *W*. *ewingi lupella* with 50/158 (32%) and *W*. *micropelta* with 40/158 (25%) were most frequent. Only 25/243 (10%) *Leptotrombidium* sp. were collected, of which 21/25 were identified as *L*. *deliense* and the remainder could not be identified to species. Nine individuals of 2 genera could only be identified to genus due to damage to key taxonomic features.

Free-living chiggers were collected at 4 sites in November 2015 and October 2016 (Figure 1). In total 163 individuals were collected and all were identified. In November 2015, 109 *Schoengastia kanhaensis* were collected along a stream, agricultural and fallow areas in Phonhong and Thoulakhom districts. In October 2016, 45 *L. deliense* were collected from a small area of 10m x 10m of shaded damp grass and leaf-litter close to a marsh in Naxaithong district. All 406 individual chiggers (n=163 were free-living and n=243 from small mammals) tested negative for *O*. *tsutsugamushi* by PCR (Table 2).

A total of 64 pools, composed of 1203 individuals were tested for *O*. *tsutsugamushi* by PCR. Pools contained mixed species. A single pool from a *B*. *indica* collected in October 2016 in Phonhong district (18.319 N, 102.469 E) tested positive. *W*. *ewingi lupella* and *W*. *micropelta* were identified on this host animal.

IgG IFA for *O*. *tsutsugamushi* was performed on 52/60 small mammals trapped in February and October 2016 in Thoulakhom and Phonhong districts. These comprised of 11 species (Table 3). Sixteen of 52 (31%) were seropositive, consisting of 5 species. In February 2016, 9/23 small mammals trapped in Thoulakhom district were seropositive from 4 species (*Niviventer* sp., *Maxomys surifer, Rattus andamanensis* and *Leopoldamys edwardsii*), whilst 1/17 (6%) (*B. indica*) from Phonhong district tested positive. In October 2016, 6/12 (50%) were seropositive, all of which were *B*. *indica*.

## Discussion

Laos carries one of the highest documented burdens of scrub typhus infection across the Asia-Pacific. Few investigations have taken place into the ecology of the disease to understand key vector species and small mammal hosts. To our knowledge, the only investigations into chiggers in Laos were performed by the Institute for Medical Research, Kuala Lumpur in the 1960s[28, 29] and in relation to the recently described Laotian rock-rat, *Laonastes aenigmanus*[30]. Here we report the first identification of *O*. *tsutsugamushi* in chiggers in Laos and a description of chigger species collected at sites across Vientiane Province, close to the capital city.

Fifteen species of small mammal were trapped, comprising 131 individuals and infested with 16 species of chigger. Of the 131 small mammals, 406 individual chiggers and 64 pools of chiggers tested, only a single chigger pool (20 individuals) was *O*. *tsutsugamushi* positive. This gives an unexpectedly low positivity rate of 1.6% of chigger pools. In previous studies, infection rates using molecular methods have ranged from 0.6% to 5% in individual chiggers, 9.6 to 56% in chigger pools and 10% of small mammals[1].

These low rates may be accounted for by several factors. A relatively low number of small mammals were trapped in this study. Rodent capture rates are known to be lower in Laos compared to neighbouring Thailand and Cambodia[31]. This observation remains difficult to explain, but may be due to high hunting intensity by local communities and that capture rates are influenced by seasonal climatic conditions and climatic variability. Two of the three collection periods took place in the dry season, during which time chigger indices and infestation rates are typically lower as a consequence of the impact of dessication on the life cycle[1], although the proportion of positives would be expected to be similar[15]. The single positive pool was collected during the rainy season, when only 16 small mammals were trapped. Of these, 15 (94%) were infested with chiggers with much higher indices, and accounted for 718/1,446 (49%) of the total collected. The proportion of recognised human vector species, such as *L*. *deliense*, was very low at 10%. A previous study in Thailand identified high risk sites for human infection to be associated with a high proportion of known human vector species (*Leptotrombidium* spp.)[15]. The reason for this is uncertain. *L*. *deliense* is a host generalist, and present in a wide range of habitats with known seasonal fluctuations[15, 32]. The factors that determine the density and diversity of different chigger species across habitats remain poorly known. The distribution of *O*. *tsutsugamushi* infection in chiggers is likely to be heterogeneous and change over time as has been described in the concept of “mite islands”[1].There is a possibility that certain habitats were under sampled due to a reliance on hunters. Rodent seropositivity at 35% was higher than that reported on average (at 30%) across all studies[1]. This suggests that despite the low rates of molecular evidence of *O*. *tsutsugamushi* in chiggers and small mammals, there is evidence of high rates of exposure to the pathogen. In small mammals the pathogen was detectable in blood for a mean of 97 days[33], until 8 weeks after the bite of an infected *Leptotrombidium* chigger[34], and in kidney tissue after 4 months[35]. In one study in Thailand IgG persisted for 10 months in animals removed from the wild and kept in captivity[36].

The lone positive chigger pool in this study consisted of mixed *Walchia* species, although the presence of *L*. *deliense* cannot be excluded as not all chiggers were identified. There is no evidence that *Walchia* acts as an *O*. *tsutsugamushi* vector for humans, but a small number of studies have identified *O*. *tsutsugamushi* in this genus[1, 15] suggesting the potential to act as an intra-zootic vector.

There are a number of limitations to this study. Firstly collections took place only at 3 time points over an 18 month period and only once (October 2016) during the wet season. In October 2016 only a small number of animals were trapped, predominantly due to unavailability of hunters due to the peak rice harvesting period. Not all rodent tissues were tested by PCR. Previous studies have demonstrated that *O*. *tsutsugamushi* positivity can vary between organ types, with spleen and lung tissue most frequently positive[15]. Seventeen percent (243/1446) of chiggers removed from small mammals were identified to species level. It is likely the overall diversity is under-represented.

## Conclusion

Despite the low rates of *O*. *tsutsutgamushi* PCR positive vectors and hosts in this study, evidence from serological data confirms the presence of the pathogen in endemic areas. Further studies are needed to identify the key vector species and ecological factors associated with high risk sites for human transmission in Laos. This will be especially important for planning the prevention and management of this neglected disease, given the evidence that the consequences of global warming, especially flooding, may increase disease burden[9].

## Figures and Tables

**Table 1:** Small mammal species, chigger infestation rates and *O*. *tsutsugamushi* PCR of organs and pools of chiggers collected from small mammals.

Small mammal species	Number	Chigger infestation (%)	*O*. *tsutsugamushi* PCR positive organs^a^	*O*. *tsutsugamushi* PCR positive chigger pools	Habitats^b^
*Bandicota indica*	33	28/33 (85)	0/33	1/33	a,b,c,f
*Berylmys berdmorei*	2	2/2 (100)	0/2	-	b,c
*Berylmys bowersi*	1	1/1 (100)	0/1	-	d
*Leopoldamys edwardsi*	4	3/4 (75)	0/4	0/1	d
*Maxomys* sp.	3	3/3/ (100)	0/3	0/7	a,d
*Maxomys surifer*	6	6/6 (100)	0/6	0/1	d
*Mus cervicolor*	2	2/2 (100)	0/2	0/1	a
*Mus caroli*	11	5/11 (46)	0/11	-	b,f
*Mus cookii*	1	1/1 (100)	0/1	-	b
*Niviventer* sp.	14	10/14 (71)	0/14	0/5	a,d
*Niviventer longbianis*	1	1/1 (100)	0/1	0/1	d
*Rattus andamanensis*	4	3/4 (75)	0/4	0/1	d
*Rattus exulans*	24	3/24 (13)	0/24	0/11	c,e
*Rattus losea*	5	2/5 (20)	0/5	0/1	a,b,f
*Rattus sakaratensis*	10	7/10 (70)	0/10	-	b
*Rattus tanezumi*	8	5/8 (63)	0/8	-	b
*Tupaia glis*	1	0/1 (0)	0/1	-	d
**Total**	**131**	**78/131 (60)**	**0/131**	**1/64**	

a PCR performed on spleen for 131 individuals and on spleen, liver and lung in 60/131 (46%) of individuals.

b a = fallow, b = rice field, c = plantation, d = secondary forest, e = village, f = water edge

**Table 2:** Chigger species collected from small mammals and free-living using the black plate method, with *O*. *tsutsugamushi* PCR results of individual chiggers.

Chigger species	Number from small mammals	Number free-living	Rodent species^a^	Season	*O*. *tsutsugamushi* PCR
*Ascoschoegastia indica*	33	-	Ms, Nsp, Ra, Re, Rt	dry & wet	0/33
*Blankaartia acuscutellaris*	13	-	Bi, Rl, Rs	dry	0/13
*Eutrombicula* sp.	-	8	-	wet	0/8
*Gahrliepia marshi*	1	-	Bbo	dry	0/1
*Gahrliepia tylana*	1	-	Bbo	dry	0/1
*Leptotrombidium deliense*	21	45	Bi, Nsp, Rs, Rt	dry & wet	0/66
*Leptotrombidium* sp.	4	-	Bi, Ms	dry & wet	0/4
*Lorillatum kianjoei*	5	-	Le, Msp, Bi	dry	0/5
*Microtrombicula chamlongi*	-	1	-	dry	0/1
*Schoengastia kanhaensis*	6	109	Bi, Rs, Rt	dry	0/115
*Schoutedenichia centralkwantunga*	1	-	Rt	dry	0/1
*Walchia alpestris*	8	-	Bbo	dry	0/8
*Walchia disparunguis disparunguis*	1	-	Rl	dry	0/1
*Walchia ewingi ewingi*	13	-	Bi, Rs	dry & wet	0/13
*Walchia ewingi lupella*	50	-	Bi, Mco, Re, Rs, Rt	dry & wet	0/50
*Walchia isonychia*	3	-	Bi, Ms	dry	0/3
*Walchia kritochaeta*	25	-	Bi, Le, Mca, Msp, Nsp, Rt	dry & wet	0/25
*Walchia micropelta*	40		Bi, Mce, Ms, Msp, Nsp, Nl, Ra, Re	dry & wet	0/40
*Walchia minuscuta*	13	-	Bbe, Bi, Ms	dry & wet	0/13
*Walchia* sp.	5		Bi, Mca, Ms, Nsp, Rs, Rt	dry & wet	0/5
**Total**	**243**	**163**			**0/406**

a Bi = *Bandicota indica*, Bbe = *Berylmys berdmorei*, Bbo = *Berylmys bowersi*, Le = *Leopoldamys edwardsi*, Msp = *Maxomys* sp., Ms = *Maxomys surifer*, Mce = *Mus cervicolor*, Mca = *Mus caroli*, Mco = *Mus cookii*, Nsp = *Niviventer* sp., Nl = *Niviventer longbianis*, Ra = *Rattus andamanensis*, Re = *Rattus exulans*, Rl = *Rattus losea*, Rs = *Rattus sakaratensis*, Rt = *Rattus tanezumi*, Tg = *Tupaia glis*

**Table 3:** Scrub typhus IgG immunofluorescent assay (IFA) results for small mammal species.

Small mammal species	Number from small mammals	Scrub typhus IgG (IFA) positive (%)
*Bandicota indica*	19	7 (37%)
*Berylmys berdmorei*	1	0
*Leopoldamys edwardsi*	4	2 (50%)
*Maxomys* sp.	6	3 (50%)
*Mus caroli*	1	0
*Mus cervicolor*	2	0
*Mus cookii*	1	0
*Niviventer* sp.	10	3 (30%)
*Rattus andamanensis*	2	1 (50%)
*Rattus losea*	5	0
*Tupaia belangeri*	1	0
**Total**	**52**	**16 (31%)**
